# DNA Binding, Photonuclease Activity and Human Serum Albumin Interaction of a Water-Soluble Freebase Carboxyl Corrole

**DOI:** 10.3390/molecules21010054

**Published:** 2015-12-31

**Authors:** Ning Na, Da-Qiang Zhao, Heng Li, Nan Jiang, Jin-Yan Wen, Hai-Yang Liu

**Affiliations:** 1 Department of Kidney Transplantation, The Third Affiliated Hospital of Sun Yat-sen University, Guangzhou 510630, China; nngg20102009@hotmail.com (N.N.); zhaodaq@mail.sysu.edu.cn (D.-Q.Z.); midget201086@gmail.com (H.L.); 2 Department of Hepatic Surgery, The Third Affiliated Hospital of Sun Yat-sen University, Guangzhou 510630, China; 3 Department of Chemistry, South China University of Technology, Guangzhou 510640, China; wjy1990scut@163.com

**Keywords:** corrole, DNA, human serum albumin, photonuclease activity

## Abstract

The DNA binding property of 5,10,15-Tris(4-carboxyphenyl) corrole (TCPC) was studied by UV-Visible, fluorescence and circular dichroism (CD) spectroscopic methods. TCPC can bind to ct-DNA via an outside binding mode with the binding constant of *K*_b_ = 1.05 × 10^5^ M^−1^. TCPC also displayed good photonuclease activity, which involves singlet oxygen species (^1^O_2_). The binding constant between TCPC and human serum albumin (HSA) is *K*_A_ = 2.24 × 10^5^ M^−1^ with a simulated binding distance of 2.06 nm. The fluorescence quenching of HSA by TCPC followed a static quenching process. Site marker competitive displacement experiments indicated that warfarin site I is the main binding site. The secondary structure of HSA was changed upon interaction with TCPC, which was confirmed by UV-Visible and CD spectroscopy.

## 1. Introduction

**Scheme 1:**
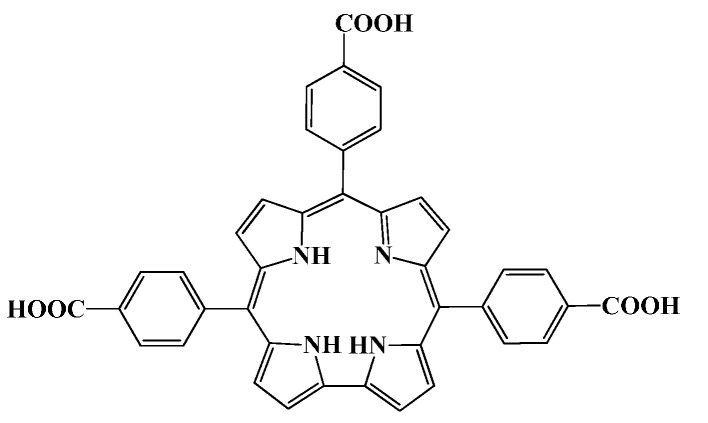
Molecular structure of 5,10,15-Tris(4-carboxyphenyl) corrole (TCPC).

Study on corrole macrocycle is one of the most active branches in modern porphyrin chemistry [1]. Water soluble or amphiphilic corrole derivatives have potential medicinal applications [2]. Water soluble pyridinium porphyrin TMPyP4 was proved to have G-terad DNA binding selectivity [3]. It has been also observed that water soluble cationic pyridinium corrole and its copper complex could stabilize DNA G-quadruplex structures [4,5] and its manganese complex even exhibited nuclease-like activity [6]. Anionic sulfonated manganese and gallium corrole complexes can bind strongly to DNA via outside binding mode and displayed oxidative and photonuclease activity [7,8]. When binding to serum albumins, anionic sulfonated manganese corrole may even show enantioselective catalytic activity in the oxidation of prochiral sulfides by H_2_O_2_ [9]. By using cell penetrating viral carrier proteins, sulfonated corrole metal complexes might be specifically delivered to tumor cell and exhibited good anticancer activity [10]. Carrier proteins were found to play an important role in the tumor detecting and eliminating by sulfonated gallium corrole [11]. Although human serum albumin (HSA) is a very important carrier protein in human blood, reports on the interaction between HSA and corrole dervatives are still less [12]. Carboxyl corrole is a kind of anionic water soluble corrole. Previously, we have reported the oxidative DNA cleavage by manganese and iron carboxyl corrole [13,14]. In this article, we wish to report the interaction of this free base carboxyl corrole (Scheme 1) with DNA and HSA at physiological conditions and its photonuclease activity.

## 2. Results and Discussion

### 2.1. DNA Binding Properties

#### 2.1.1. Electronic Spectroscopy

**Figure 1:**
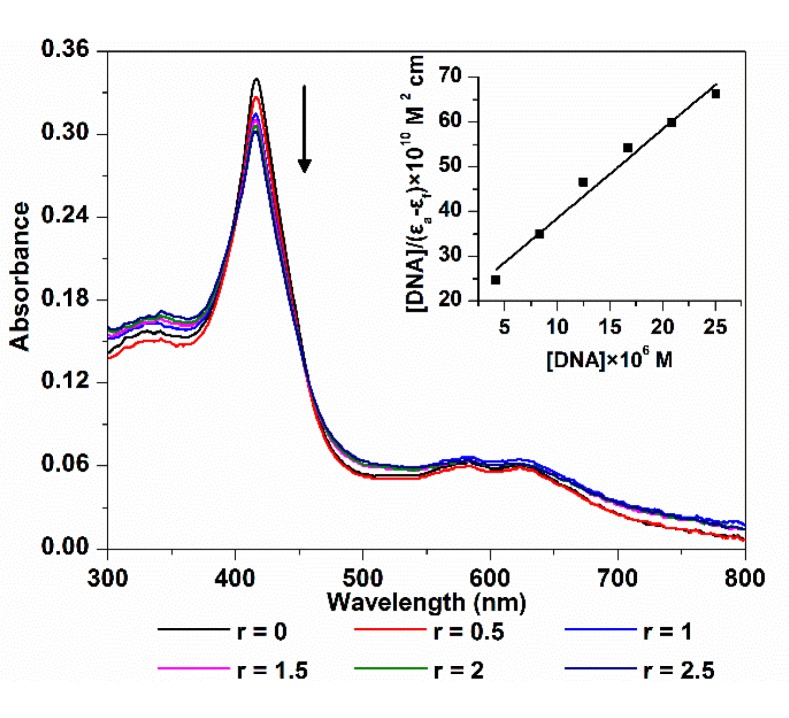
Electronic spectra changes of TCPC (10 μM) upon addition of ct-DNA (*r* = [DNA]/[TCPC] = 0, 0.5, 1, 1.5, 2, 2.5) in Buffer I; the inset is the plot of [DNA]/|(ε_a_ − ε_f_)|* vs.* [DNA].

The electronic spectra of 5,10,15-Tris(4-carboxyphenyl) corrole (TCPC) in the absence and presence of ct-DNA are shown in Figure 1. The absorption intensity of TCPC was gradually decreased upon the addition of ct-DNA, and yielded an 11.1% hypochromism with negligible wavelength shift. Generally, there are three major interaction modes between small molecules and DNA, intercalation, outside groove binding and outside binding with self-stacking. For DNA-porphyrinoids system [15], intercalation interaction is characterized by an obvious red shift (≥15 nm) and a large hypochromism (≥35%) of soret band, the outside groove binding is characterized by no (or minor) red shift (≤8 nm) and smaller hypochromic effect (≤10%), a red or blue shift (for J- and H-aggregates, respectively) and moderate hypochromism elucidate aggregation. The current observed electronic spectra changes may be interpreted as the results of an outside binding between TCPC and ct-DNA, which is similar to its manganese and iron complex [13,14] and its porphyrin analogue [16]. The intrinsic-binding constant *K*_b_ of TCPC and ct-DNA might be obtained using the following Equation [17]:

(1) $$\frac{\left[\text{DNA}\right]}{\left|\left({\text{ε}}_{\text{a}}-{\text{ε}}_{\text{f}}\right)\right|}=\frac{\left[\text{DNA}\right]}{\left|\left({\text{ε}}_{\text{b}}-{\text{ε}}_{\text{f}}\right)\right|}+\frac{1}{K_{\text{b}}\left|\left({\text{ε}}_{\text{b}}-{\text{ε}}_{\text{f}}\right)\right|}$$

where ε_a_, ε_f_ and ε_b_, are apparent, free and bound complex extinction coefficients, respectively. [DNA] is the concentration of DNA in the base pairs. From the fit plot of [DNA]/(ε_a_ − ε_f_) *vs.* [DNA], *K*_b_ was given by the ratio of slope to the intercept. The calculated binding constant (*K*_b_) was 1.05 × 10^5^ M^−1^, which is larger than that of sulfonated corrole (1.85 × 10^4^ M^−1^) [8], indicating higher affinity between TCPC and DNA.

#### 2.1.2. Fluorescence Spectroscopy

**Figure 2:**
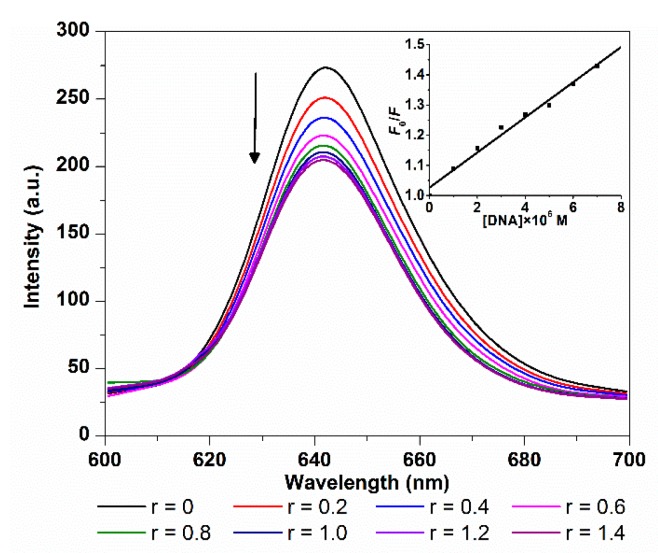
Fluorescence spectra changes of TCPC (5 μM) on the titration of increasing amounts of ct-DNA(*r* = [DNA]/[TCPC]) in Buffer I (λ_ex_ = 420 nm); the inset is the plot of *F*_0_/*F vs.* [DNA].

The changes in fluorescence emission spectra of TCPC with addition of ct-DNA are shown in Figure 2. As the amount of ct-DNA increased, an obvious decrease in emission intensity was observed. The quenching extent is similar to those outside binding molecules such as sulfonated corrole [7,8] and carboxyl porphyrin [16]. The fluorescence quenching of TCPC by ct-DNA can be evaluated by classical Stern–Volmer equation [18,19]:

(2) *F*_0_/*F* = 1 + *K*_SV_[Q] = 1 + *K*_q_τ[Q]

where *F*_0_ and *F* are the fluorescence intensity in the absence and presence of quencher, respectively. τ(~10^−8^ s) is the lifetime of the fluorophore and [Q] is the concentration of quencher. *K*_SV_ and *K*_q_ are the Stern–Volmer quenching constant and quenching rate constant, which were obtained from the slope of *F*_0_/*F* *vs.* [Q] linear plot (Figure 2 inset). The good linear quenching plot and large *K*_q_ (5.86 × 10^12^ M^−1^·s^−1^) illustrated that the TCPC fluorescence quenching by DNA was a static process, and the calculated *K*_SV_ value was 5.86 × 10^4^ M^−1^, indicating a strong quenching efficiency. The *K*_SV_ and *K*_q_ of TCPC and ct-DNA binding are comparable to those of sulfonated iron corrole complex (*K*_SV_ = 1.13 × 10^5^ M^−1^; *K*_q_ = 1.13 × 10^13^ M^−1^·s^−1^) [20].

#### 2.1.3. Circle Dichroism Spectroscopy

The B form DNA shows two characteristic circular dichroism (CD) bands in the UV region: a positive band at 275 nm due to base stacking and a negative band at 245 nm due to polynucleotide helicity [21]. The conformational variations of nucleic acids induced by the interaction with small molecules can be sensitively monitored by CD spectroscopy [22]. CD spectra changes of DNA can provide useful information about the interaction mode between probe molecules and DNA. Simple electrostatic or groove binding shows less perturbation of the base stacking and helicity bands, while an intercalative interaction will enhance the intensities of both CD bands significantly. Moreover, the interaction of porphyrinoids with DNA may lead to an induced CD (ICD) at the porphyrin Soret band, which can be used to also estimate the binding mode: a negative ICD band is characteristic of intercalation, a positive band is signal of external (minor groove) binding, and no obvious ICD band could be interpreted as outside binding mode [23].

**Figure 3:**
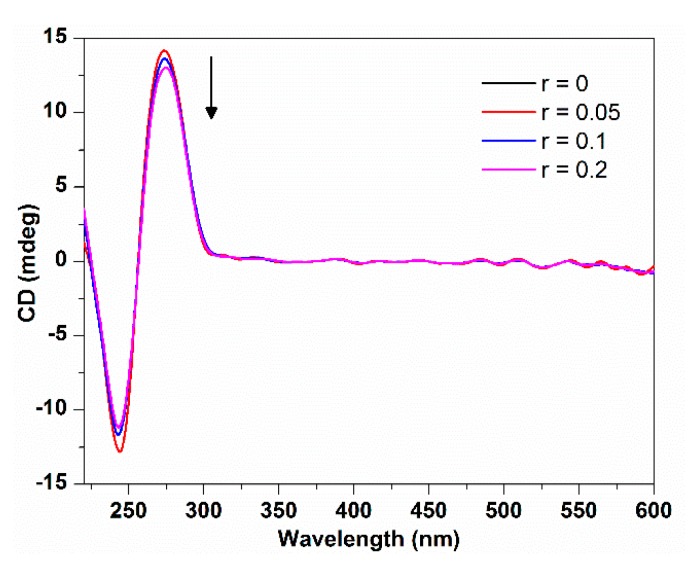
CD spectra changes of ct-DNA (150 μM) with the addition of TCPC (*r* = [TCPC]/[DNA] = 0, 0.05, 0.1, 0.2) in Buffer I.

Figure 3 shows the CD spectra changes of ct-DNA upon the addition of TCPC. With the increasing TCPC concentration, both positive and negative peaks decreased in ellipticity slightly with no obvious wavelength shift. This indicated that the interaction between them does not perturb the conformation of ct-DNA significantly. The small changes in ct-DNA characteristic CD bands and no ICD at the Soret band provided supplemental evidence of outside binding mode between TCPC and ct-DNA.

#### 2.1.4. Viscosity Measurements

**Figure 4:**
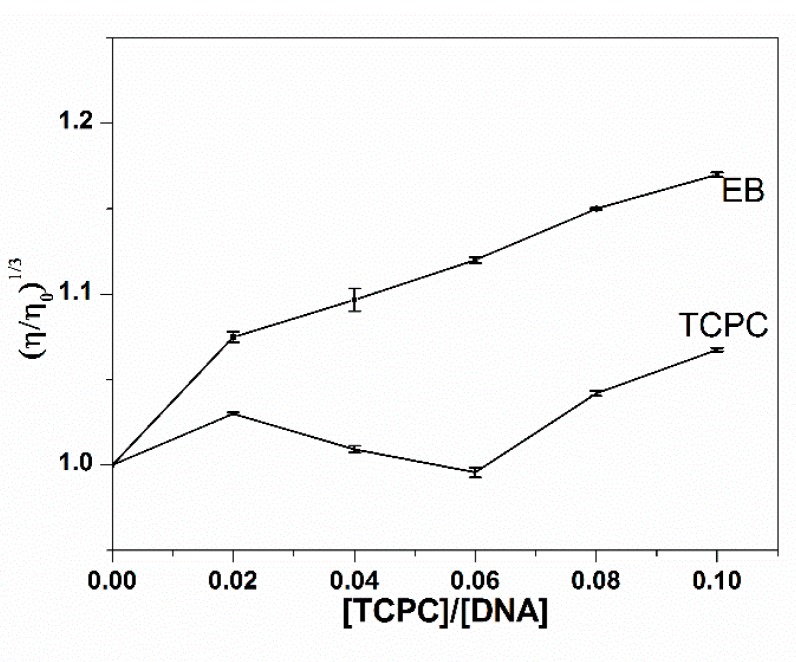
Effect of the addition of TCPC on the relative viscosities of DNA (100 mM) in Buffer I at 30 ± 0.1 °C.

Viscosity measurements, sensitive to the length of DNA, were often used to investigate the binding mode of small molecules and DNA. An intercalative interaction generally elongates the DNA helix to accommodate the binding ligand and increase the DNA viscosity, while non-classical or partial intercalation could bend or kink the DNA helix, causing the decrease of viscosity. An outside groove binding has almost no effect on DNA length or viscosity under the same condition. As can be seen in Figure 4, there were no significant changes in the relative viscosity of ct-DNA upon the addition of TCPC, suggesting an outside binding mode between TCPC and DNA, which is consistent with the spectral results.

### 2.2. DNA Photocleavage

**Figure 5:**
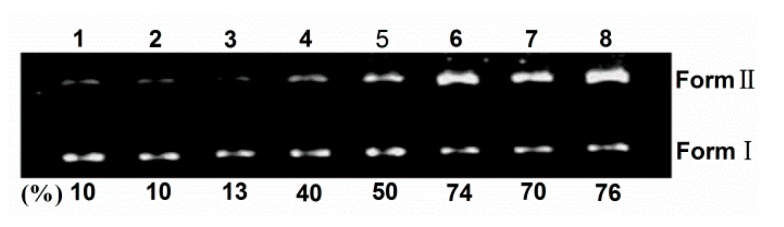
Photocleavage of pBR322 DNA (0.1 μg) by various concentrations of TCPC in Buffer II (pH = 7.2). lane 1, DNA alone (light); lane 2, DNA alone (dark); lane 3, DNA + 80 μM TCPC (dark); lane 4, DNA + 20 μM TCPC; lane 5, DNA + 40 μM TCPC; lane 6, DNA + 80 μM TCPC; lane 7, DNA + 120 μM TCPC; lane 8, DNA + 160 μM TCPC.

Sulfonated and hydroxyl corrole can efficiently cleave DNA at low concentration under illumination, and hydroxyl radical or singlet oxygen were found as the active intermediate [8,24]. Here, the photoactivated DNA-cleavage activity of TCPC was monitored by agarose gel electrophoresis. Figure 5 shows the gel electrophoresis results of pBR322 DNA after incubation with TCPC. No significant cleavage was observed for control lanes in which TCPC was absent (lane 1–2) or incubation of the plasmid DNA with TCPC in the dark (lane 3). Under irradiation, the amount of Form I DNA was gradually diminished with increased concentrations of TCPC (lanes 4–8), and Form II DNA increased. Hence, TCPC exhibits photo- and concentration-dependent single-strand DNA cleavage activity.

**Figure 6:**
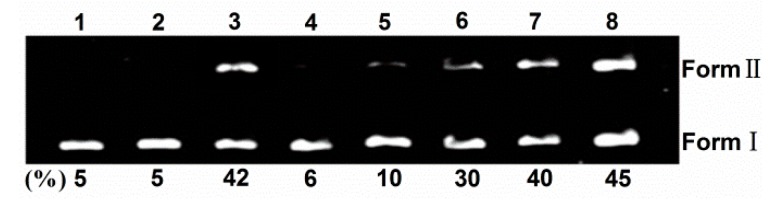
Photocleavage of pBR322 DNA (0.1 μg) by TCPC (40 μM) in the presence of additives in Buffer II (pH = 7.2). lane 1, DNA alone; lane 2, DNA + 40 μM TCPC (dark); lane 3, DNA + 40 μM TCPC; lane 4, DNA + 40 μM TCPC + 100 mM NaN_3_; lane 5, DNA + 40 μM TCPC + 100 mM DABCO; lane 6, DNA + 40 μM TCPC +100 mM KI; lane 7, DNA + 40 μM TCPC + 500 μM DMSO; lane 8, DNA + 40 μM TCPC + 500 μM *tert*-BuOH.

To shed light on the nature of reactive species that are responsible for the photo-activated cleavage of the plasmid DNA by TCPC, further investigation on the influence of different potentially inhibiting agents were performed (Figure 6). With the existence of singlet oxygen (^1^O_2_) scavengers (lanes 4–5), the activity of TCPC was inhibited, suggesting the possible involvement of ^1^O_2_ in the DNA photocleavage. While the addition of hydroxyl radical (·OH) scavengers (lanes 7–8) had no apparent effect on the DNA cleavage efficiency. Noteworthy, KI is an often-used hydroxyl radical (OH) scavenger. However, it could reduce the DNA cleavage efficiency obviously (lane 6). This may be rationalized by the oxidation of KI by singlet oxygen. These observations suggest ^1^O_2_ is responsible for the DNA photocleavage by TCPC.

### 2.3. HSA Binding Behavior

#### 2.3.1. HSA–TCPC Fluorescence Characteristics

Interactions with molecules can give rise to the fluorescence quenching of host molecules through energy transfer, excited state reactions, the formation of ground-state complex, collision, *etc.* [25]. Generally, the fluorescence of protein comes from tryptophan (Try), tyrosine (Tyr) and phenylalanine (Phe) exists. Due to the low quantum yield of phenylalanine and the fluorescence quenching of tyrosine when it is ionized or close to amino groups, carboxyl groups [26],the single tryptophan is responsible for the majority of the intrinsic fluorescence of the protein. The fluorescence emission of protein is sensitive to the changes in the local environment of the tryptophan and so can be attenuated by binding a small molecule at or near this residue [27].

**Figure 7:**
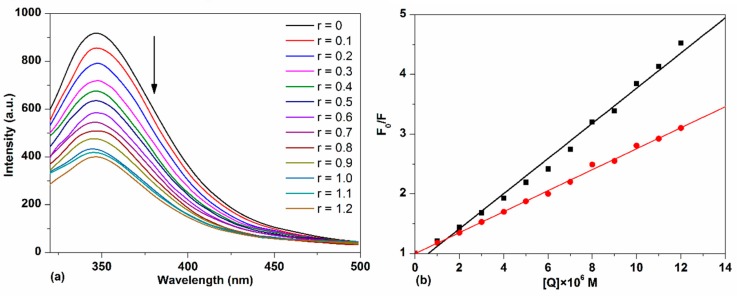
(**a**) Fluorescence spectra changes of HSA with the addition of TCPC (*r* = [TCPC]/[DNA]) in Buffer IV (pH = 7.4); (**b**) the plots of *F*_0_/*F* *vs.* [TCPC] before (**darkline**) and after (**red line**) IFE (inner filter effect) correction.

The emission spectra of HSA with different concentrations of TCPC are shown in Figure 7a. It can be observed that HSA exhibited a strong fluorescence emission peak at 346 nm after being excited at 295 nm. With the addition of TCPC, a progressive decrease in fluorescence intensity was observed. Considering TCPC has significant absorbance at 295 nm and 346 nm, which is near the excitation and emission maximum of HSA florescence spectra. The inner filter effect must be considered when doing the quenching constant calculations by the Stern–Volmer equation. The correction factor for the effect at each TCPC concentration is calculated according to the following equation [28]:

(3) $$\frac{F_{\text{cor}}}{F_{\text{obsd}}}=\frac{2.3dA_{\text{ex}}}{1-10^{-dA_{\text{ex}}}}10^{gA_{\text{em}}}\frac{2.3sA_{\text{em}}}{1-10^{-sA_{\text{em}}}}$$

where *F*_obsd_ is the observed intensity, *F*_cor_ is the corrected intensity, and *A*_ex_ and *A*_em_ are the absorbance per centimeter at the excitation and emission wavelengths, respectively. *d* is the width of the cuvet used during the measurement, *s* is the thickness of the excitation beam, and *g* is the distance between the edge of the beam to the edge of the cuvette.

The fluorescence quenching efficiency of HSA by TCPC before and after IFE correction is shown in Figure 7b. Obviously, the weaker quenching efficiency with IFE correction was observed and the corrected calculation results are listed in Table 1. The good linear fit of the Stern–Volmer plot generally indicates a single class of fluorophore in a protein, this also means that only one quenching process (dynamic or static) occurs in this case [29]. For a dynamic process, the maximum diffusion collision quenching rate constant (*K*_q_) of various complexes with protein is 2 × 10^10^ M^−1^·s^−1^, and a higher quenching rate constant means a static process [19]. The value of *K*_q_ obtained (~10^13^ M^−1^·s^−1^) is much larger than 2 × 10^10^ M^−1^·s^−1^, suggesting a static fluorescence quenching of HSA by TCPC with the formation of TCPC-HSA complex.

**Table 1:** Binding parameters of competitive experiments of 5,10,15-Tris(4-carboxyphenyl) corrole-human serum albumin (TCPC-HSA) system (T = 298 ± 0.1 K).

Site Marker	*K*_SV_ × 10^−5^ (M^−1^)	*K*_q_ × 10^−13^ (M^−1^·s^−1^)	*K*_A_ × 10^−5^ (M^−1^)	*n*
blank	1.75	1.75	2.24	1.14
ibuprofen	1.23	1.23	1.62	1.23
warfarin	0.98	0.98	1.21	1.36

For a static quenching process, the association constant (*K*_A_) and the average number of binding site (*n*) can be calculated using Equation (4) [30]:

(4) $$\text{log}\left\{\left(F_{0}-F\right)/F\right\}=nlogK_{\text{A}}-nlog\left\{1/\left[{\text{Q}}_{\text{t}}\right]-\left(F_{0}-F\right)\left[{\text{P}}_{\text{t}}\right]/F_{0}\right\}$$

where *F*_0_ and *F* are the fluorescence intensities of HSA in the absence and presence of quencher, [Q_t_] and [P_t_] are the total concentrations of the complex and HSA, respectively. The calculated association constant is on the order of 10^5^ M^−1^ for TCPC (Table 1), manifesting strong binding to the protein.

#### 2.3.2. Site-Selective Binding of TCPC on HSA

**Figure 8:**
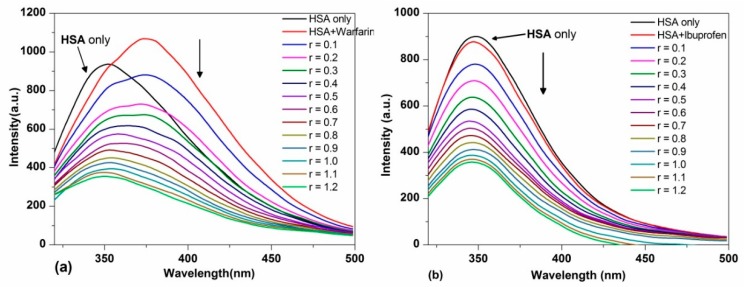
Fluorescence spectra changes of HSA (5 μM) in the presence of equimolar warfarin (**a**) and HSA-ibuprofen (**b**) in Buffer IV (pH = 7.4) solution (Pre-incubated for 5 min) with the addition of TCPC (*r* = [TCPC]/[HSA]).

In order to identify the binding site of TCPC on HSA, the site marker competitive experiments were performed using warfarin and ibuprofen as site marker fluorescence probes for monitoring site I (Subdomain IIA) and site II (Subdomain IIIA) of HSA, respectively [31,32]. As shown in Figure 8a, with the addition of warfarin to the HSA solution, the maximum emission wavelength of HSA showed an obvious red shift, and the intensity was significantly increased. However, for ibuprofen, the fluorescence maximum of the ibuprofen-HSA system was nearly the same as HSA (Figure 8b).

**Figure 9:**
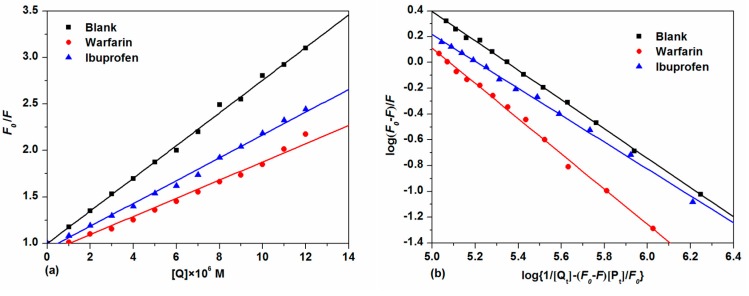
Plots of *F*_0_/*F* *vs.* [Q] (**a**) and log(*F*_0_ − *F*)/*F* *vs.* log{1/[Q_t_] − (*F*_0_ − *F*)[P_t_]/*F*_0_}; (**b**) for competitive experiments of TCPC and HSA-site marker systems.

With the further addition of TCPC, the fluorescence intensity of HSA with both site makers underwent a distinguished decrease. For the warfarin-HSA system, an obvious blue shift was also observed. To facilitate the comparison of the influence of warfarin and ibuprofen on the interactions between TCPC and HSA, the binding constants in the presence of site markers were analyzed using Equation (2) (Figure 9a) and Equation (4) (Figure 9b), respectively. The results were listed in Table 1. Obviously, the binding constants of TCPC-HSA at the presence of site markers are less than those without markers. Moreover, in the presence of warfarin, the binding constants reduced more significantly. A plausible explanation for the results is that TCPC can bind at both site I and site II, while site I is the primary binding site for TCPC [33].

#### 2.3.3. Energy Transfer between TCPC and HSA

**Figure 10:**
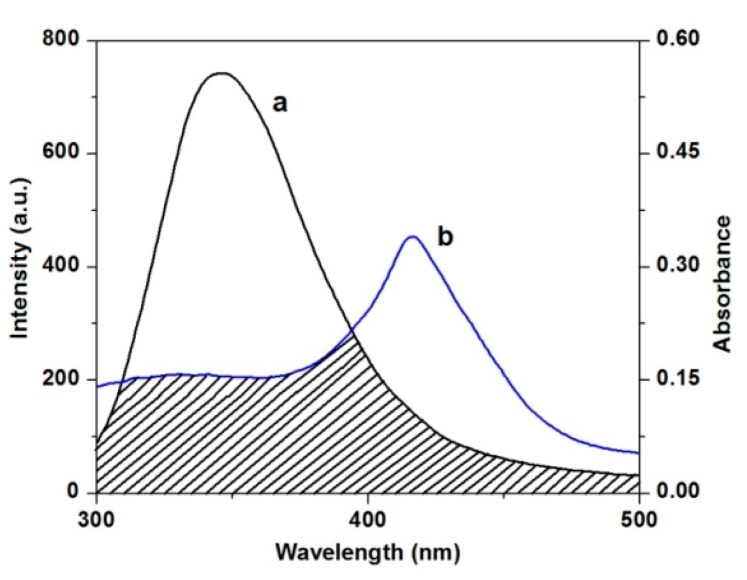
Spectra overlap of the fluorescence spectra of HSA (**a**) with the absorption spectra (**b**) of TCPC.

Fluorescence resonance energy transfer (FRET) is a distance dependent process between different electronic excited states of molecules in which excitation energy is transferred from one molecule (donor) to another molecular (acceptor) without emission of a photon from the former molecule system [34]. FRET has been used for measuring molecular distances in biological and micromolecular systems. The spectral overlap between the absorption spectrum of TCPC (10 μM) and the fluorescence emission spectrum of HSA (10 μM) is shown in Figure 10. According to FRET theory [34], the efficiency of energy transfer E can be calculated by Equation (5):

(5) $$\text{E}=1-F/F_{0}=R_{0}^{6}/\left(R_{0}^{6}+r^{6}\right)$$

where *F* and *F*_0_ are the fluorescence intensity of HSA in the presence and absence of TCPC, *r* is the distance between acceptor and donor, and *R*_0_ is the critical distance when the efficiency of transfer is 50%. The value of *R*_0_ was evaluated using the equation:

(6) $$R_{0}^{6}=8.79\times 10^{-25}K^{2}n^{-4}\text{Φ}J$$

In Equation (6), *K*^2^ is the orientation factor related to the geometry of the donor and acceptor of the dipoles and *K*^2^ = 2/3 for random orientation as in fluid solution; *n* is the average refracted index of medium, Φ is the fluorescence quantum yield of the donor, and *J* is overlap integral of the fluorescence emission spectrum of donor and absorption spectrum of the acceptor, which is given by Equation (7):

(7) $$J\left(\text{λ}\right)=[\overset{\infty }{\underset{0}{\int }}F\left(\text{λ}\right)\text{ε}\left(\text{λ}\right){\text{λ}}^{4}d\text{λ}]/[\overset{\infty }{\underset{0}{\int }}F\left(\text{λ}\right)\text{dλ}]$$

where *F*(λ) is the corrected fluorescence intensity of donor in the wavelength range, from λ to λ + Δλ, ε(λ) is the extinction coefficient of the acceptor at λ. For the ligand-HSA interaction, *n* = 1.336, Φ = 0.118 [35].According to Equations (5)–(7), the values of the parameters were obtained to be *J* = 2.96 × 10^−14^ cm^3^·L·mol^−1^, *R*_0_ = 2.00 nm, E = 0.46, and *r* = 2.06 nm. The values of *R*_0_ and *r* in the present study are less than 10 nm, and 0.5*R*_0_ < *r* < 1.5*R*_0_, revealing that the energy transfer from HSA to TCPC occurred efficiently and the static quenching interaction was produced between them [36].

#### 2.3.4. HSA Conformation

**Figure 11:**
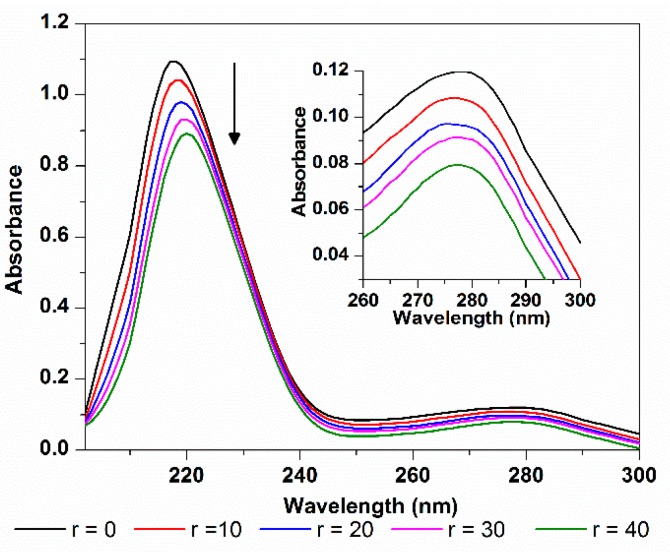
Absorption spectra changes of HSA with the addition of TCPC (*r* = [TCPC]/[HSA]) in Buffer IV (pH = 7.4).

Absorption spectroscopy can be used to explore the structure changes of protein. The absorption spectra of HSA in the absence and presence of TCPC were shown in Figure 11. HSA has two absorption peaks, the strong absorption peak at about 213 nm reflects the framework conformation of the protein, and the weak absorption peak at about 279 nm comes from the aromatic amino acids (Trp, Tyr, and Phe) [33]. When TCPC was added, the absorption spectrum of HSA showed a hypochromic effect around 213 nm with concomitant bathochromic shift, this may be attributed to the disturbance of the α-helix of protein by specific interaction with TCPC [37]. Meanwhile, the subtle change at around 279 nm reflects that the microenvironment of Trp, Tyr, and Phe aromatic acid residues was slightly altered.

**Figure 12:**
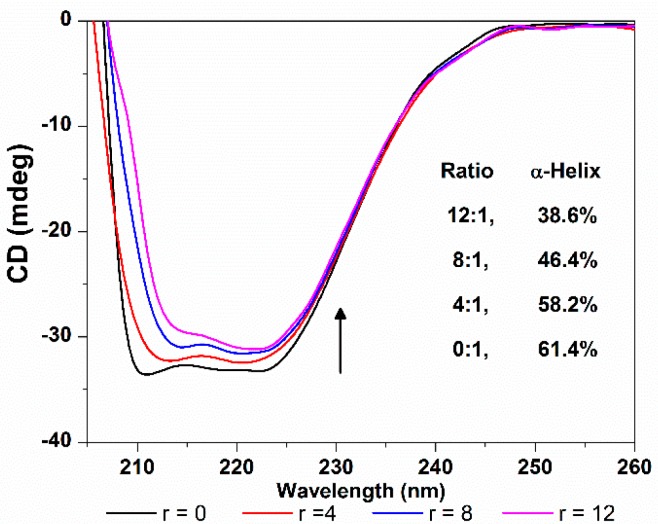
CD spectra changes of HSA with the addition of TCPC (*r* = [TCPC]/[HSA]) in Buffer IV (pH = 7.4).

CD spectroscopy is another ideal technique for monitoring the conformation changes of protein. As shown in Figure 12, the CD spectra of HSA exhibit two negative ellipticities in the ultraviolet region at round 208 and 218 nm, which is typical characterization of the α-helix structure of proteins, and both arise from the *n*-π* transition of the peptide bond of the α-helical structure [38]. The increasing of the molar ratio of TCPC to HSA (from 0:1 to 12:1) caused a decrease in CD band intensity, indicating that the interaction between TCPC and HSA induced a decrease in the helical structure content of HSA. The quantitative analysis of α-helix percentage was obtained by the following equations [39]:

(8) α-helix (%) = 100 × (−MRE − 4000)/(33000 − 4000)

(9) MRE = Observed CD (m degree)/(10*nlC*_p_)

where MRE is the observed MRE value at 208 nm, *n* is the number of amino acid residues which is 585 for HSA, and *l* is the path length (1 cm), and *C*_p_ is the molar concentration of HSA. The results showed that the α-helix content of HSA reduced from 61.4% to 38.6% upon binding TCPC. That is, the binding of TCPC to HSA may induce conformational changes of HSA characterized by the reduction of its α-helix contents.

## 3. Experimental Section

### 3.1. Materials and Methods

Calf thymus DNA (ct-DNA), HSA and ibuprofen were purchased from Sigma-Aldrich China (Shanghai, China), pBR322 DNA from Shanghai Sangon Company (Shanghai, China), and warfarin from Chengdu Aikeshiji Company (Chengdu, China). All other analytical grade reagents, Tris (Hydroxymethyl)-aminomethane (Tris-Base), sodium chloride, boric acid, dimethyl sulfoxide (DMSO), agarose gel loading buffer, and ethyldiaminetetraacetic acid (EDTA) were obtained from commercial sources, and used as received. Doubly distilled deionized water was used throughout. Buffer I, 5 mM Tris-HCl/50 mM NaCl in deionized water (pH = 7.2), was used for preparing stock solution of DNA, absorption titrations, fluorescence studies, viscosity experiments and circular dichroism detecting. Buffer II, 50 mM Tris-HCl/18 mM NaCl in deionized water (pH = 7.2), Buffer III, Tris-boric acid–EDTA (89 mM Tris, 89 mM H_3_BO_3_, 20 mM EDTA, pH = 8.3) in aqueous solution, both of the two buffer solutions were used for gel electrophoresis experiments. Buffer IV, 50 mM Tris-HCl/150 mM NaCl in deionized water (pH = 7.4), was used for all experiments concerned with HSA.

The stock solution of DNA was prepared in Buffer I, which gave the ratio of UV absorbance at 260 and 280 nm, A_260_/A_280 _= 1.8–1.9, indicating that the DNA was sufficiently free of protein [40], and concentration was determined by the intensity of UV spectrum at 260 nm with a molar extinction coefficient value of 6600 M^−1^·cm^−1^ [41].

The stock solution of HSA was prepared by dissolving the solid HSA in Buffer IV, and the concentration was determined spectroscopically by using the molar extinction coefficient (ε= 35700 M^−1^·cm^−1^ at 280 nm) [42]. All stock solutions were stored at 4 °C and used in a week.

UV-Visible spectra were measured on Hitachi 3900H UV-Vis Spectrometer (Hitachi Limited, Tokyo, Japan) and emission spectra were recorded using Perkin Elmer LS55 fluorescence spectrophotometer (Perkin Elmer, Los Angeles, CA, USA) with a 1 cm quartz cell. CD spectra were monitored on a JASCO-J810 spectrometer (JASCO, Tokyo, Japan). The viscosity experiments were carried out by using an Ubbelodhe viscometer (Hangzhou Zhongwang Technology Co., Ltd., Hangzhou, China). Gel electrophoresis was run on a DYCP-31CN electrophoresis cell produced by Beijing Liuyi instrument factory (Beijing Liuyi instrument factory, Beijing, China) and then analyzed using Gel Doc XR system (Bio-Rad, Beijing, China).

### 3.2. Synthesis of Carboxyl Corrole

5,10,15-tris(4-carboxymethylphenyl) corrole (TCPC) was synthesized according to previous procedure [13,14]. TCPC was prepared using the following procedure: TCPC (50 mg, 0.07 mmol) was dissolved in THF/CH_3_OH (100 mL, 1:2) and 6 mL aqueous KOH (2 M) was added. The mixture was stirred at 40 °C under atmosphere. Reaction was monitored by TLC, the disappearance of the starting material corrole indicates that hydrolysis reaction was complete. After cooling down to room temperature, the reaction mixture was acidified with aqueous HCl (2 M) and the resulting solution was extracted with THF/CH_2_Cl_2_ (1:1). The organic phase was collected and washed with water for three times, dried over Na_2_SO_4_, filtered, and the solvent was evaporated under reduced pressure. The crude product was recrystallized from acetone and hexane (1/5 *v*/*v*). The characterization data for the compound are given below.

TCPC: [Yield: 87%]. Analysis: Calculated for C_40_H_26_N_4_O_6_: C, 72.94; H, 3.98; N, 8.51. Found: C, 72.75; H, 3.95; N, 8.54.UV-Vis (λ_max_/nm, Buffer I): 421, 583, 625, 660 nm; ^1^H-NMR (400 MHz, D_6_-DMSO, δ, ppm):13.16 (s, 3H), 9.10 (s, 2H), 8.85 (s, 2H), 8.47–8.36 (s, 12H), 8.2 (s, 2H), 8.15 (s, 2H); ESI-MS (CH_3_OH, negative): *m/z* 765 [M + 2K + CH_3_OH-3H]^−^.

### 3.3. DNA Binding Experiments

Absorbance titration experiments were recorded at 300–800 nm by keeping the concentration of TCPC (10 μM) in Buffer I with the increasing concentration of DNA (from 0 to 25 μM). Fluorescence measurement was carried out by adding increasingly DNA (from 0 to 7 μM) into TCPC solution (5 μM) upon excitation at 420 nm in Buffer I. Each solution was kept for 10 min at room temperature to achieve equilibrium before detecting.

The CD spectra of DNA (150 μM) with different concentrations of TCPC (*r* = [TCPC]/[DNA] = 0, 0.05, 0.1, 0.2) in Buffer I were recorded at 25 °C using a 1 cm quartz cell. Each sample was mixed and equilibrated for 10 min before measurement and scanned ranging from 600 to 200 nm at the speed of 200 nm/min, and the results were obtained after averaging three scans and automatically subtracting the buffer background.

The viscosities of DNA in the presence of different concentrations of TCPC ([TCPC]/[DNA] = 0, 0.02, 0.04, 0.06, 0.08, 0.1) were measured using an ubbelodhe viscometer, which is maintained in a thermostat water bath at 30.0 ± 0.1 °C. The flow time was recorded with a digital stopwatch, each measurement was replicated for five times, and the average flow time was obtained. The viscosity values (η) were calculated by the method described in [7].

### 3.4. DNA Cleavage Experiments

The photocleavage capacity of TCPC to supercoiled DNA was determined by agarose gel electrophoresis in a total volume of 10 μL in 0.5 mL, Eppendorf microcentrifuge tubes containing 0.1 μg DNA and various concentrations of TCPC. The solutions were irradiated at room temperature with an afilament lamp for 4 h, and then quenched by adding 2 μL 0.25% bromophenol blue solution (containing 0.25% xylene cyanol FF in 60% glycerol). Electrophoresis was carried out for 2 h at 70 V on 1.1% agarose gel in Buffer III, and the resulted gel was analyzed after stained with a 1 mg/L EB solution.

The DNA photocleavage mechanism was investigated by adding several typical reactive oxygen species scavengers. Hydroxyl radical scavenger (DMSO, *tert*-BuOH and KI) and singlet oxygen quencher (NaN_3_ and DABCO) were tested. The additives were added prior to TCPC solution without incubation interval. After the addition of TCPC, the mixture incubated for 3 min. All tested additives are not photosensitizers themselves.

### 3.5. HSA Binding Experiments

Fluorescence measurement was carried out in the range of 315–500 nm after being excited at 295 nm in Buffer IV. The concentration of HSA (5 μM) was kept constant, TCPC was added from 0 to 12 μM, and each sample was performed after equilibrated for 10 min. In the binding site competitive experiment, TCPC was gradually added to the mixture of HSA and the site marker, in which the concentration ratio of HSA to the site marker was 1:1. The fluorescence intensities were recorded in the range of 300–500 nm with an excitation wavelength of 295 nm.

Absorption spectra were recorded in the range of 200–350 nm with the concentration ratio of 0, 10, 20, 30, 40 (TCPC/HSA) in Buffer IV. The CD spectra of HSA (0.4 μM) in the absence and presence of TCPC (0, 4, 8 and 12 μM) were recorded at 25 °C in Buffer IV in the range of 200–260 nm using a 1 cm quartz cell.

## 4. Conclusions

In this article, we have reported the spectroscopic investigation on interactions of a water-soluble carboxyl corrole (5,10,15-Tris(4-carboxyphenyl) corrole, (TCPC)) with DNA and HSA. The results suggested that TCPC interacts with DNA viaan outside binding mode. This corrole also exhibited good DNA cleavage activity upon illumination, and the singlet oxygen (^1^O_2_) was the active species responsible for the cleavage. In addition, TCPC displayed high affinity with HSA and mainly bound to Subdomain IIA of this protein carrier. The fluorescence of HSA may be effectively quenched by TCPC via a static process, that is via the formation of TCPC-HSA adduct. The calculated FRET binding distance *r* is 2.06 nm, and such a short distance means the energy transfer from HSA to TCPC is of high possibility. Furthermore, UV-Visible and CD spectroscopic evidence indicated that TCPC may induce conformational changes in HSA upon interaction. As compared to other corrole macrocycles, the anionic water soluble corrole TCPC may have the advantage to permeate cells more easily. The present work implies that TCPC may be used as a new photosensitizer for photodynamic therapy. The investigation of photo-cytotoxicity of TCPC towards different tumor cells is currently going on in our laboratory.
